# AAM-B Interacts with Nonstructural 4B and Regulates Hepatitis C Virus Propagation

**DOI:** 10.1371/journal.pone.0132839

**Published:** 2015-07-17

**Authors:** Eun-Mee Park, Yun-Sook Lim, Byung-Yoon Ahn, Soon B. Hwang

**Affiliations:** 1 National Research Laboratory of Hepatitis C Virus and Ilsong Institute of Life Science, Hallym University, Anyang, Korea; 2 School of Science and Biotechnology, Korea University, Seoul, Korea; Pohang University of Science and Technology, REPUBLIC OF KOREA

## Abstract

Hepatitis C virus (HCV) usurps host cellular lipid metabolism for production of infectious virus particles. Recently, we have screened a siRNA library targeting host factors that control lipid metabolism and lipid droplet (LD) formation in cell culture grown HCV (HCVcc)-infected cells. Of 10 final candidates, we selected the gene encoding AAM-B for further characterization. We showed that siRNA-mediated knockdown of AAM-B impaired HCV propagation in Jc1-infected cells. More precisely, knockdown of AAM-B abrogated production of infectious HCV particles in both Jc1 RNA electroporated cells and Jc1-infected cells. It is worth noting that knockdown of AAM-B exerted no effect on lipid droplet formation. Moreover, AAM-B interacted with nonstructural 4B (NS4B) through the C-terminal region of NS4B. Protein interplay between AAM-B and NS4B was verified in the context of HCV replication. Using either transient or stable expression of AAM-B, we verified that AAM-B colocalized with NS4B in the cytoplasm. Immunofluorescence data further showed that AAM-B might be involved in recruitment of NS4B to sites in close proximity to LDs to facilitate HCV propagation. Collectively, this study provides new insight into how HCV utilizes cellular AAM-B to facilitate viral propagation.

## Introduction

Hepatitis C virus (HCV) is a major etiologic agent of chronic liver disease [1]. Chronic HCV infection leads to severe liver diseases, including liver cirrhosis and hepatocellular carcinoma. Approximately 170 million people are chronically infected with HCV worldwide [2]. HCV is an enveloped virus with a positive sense, single-stranded RNA that belongs to the genus *Hepacivirus* in the family *Flaviviridae* [3]. HCV has been classified into 7 major genotypes and each genotype is divided into many subtypes. The HCV genome consists of 9.6 kb in length and encodes a 3,010-amino-acid protein from a single open reading frame. This polyprotein is processed by host cellular and viral proteases into 3 structural (core, E1, and E2) and 7 nonstructural (p7 and NS2 to NS5B) proteins [4]. Nonstructural 4B (NS4B) is a hydrophobic 27-kDa protein located in ER membrane [5]. NS4B has four transmembrane domains with the N and C termini located in the cytoplasm. NS4B induces the formation of the membranous web with specific single membrane vesicles. NS4B functions as a scaffold for the HCV replication complex [6–8].

Cellular lipid plays a crucial role in the HCV life cycle contributing to assembly, release, and infectivity of HCV [9]. Lipid droplet (LD), organelle that stores neutral lipids, is essential for the production of HCV particles [10]. The surface of LD is coated with a variety of proteins which plays an important role in the regulation of LD maintenance and function [11–12]. It has been reported that Rab18 interacts with HCV NS5A and regulates HCV replication and possibly production of infectious HCV particles by promoting the physical interaction between the HCV and LD [13]. TIP47 also associates with LD and regulates HCV replication [14–16]. AAM-B is a 28-kDa integral membrane protein anchored in ER membrane. AAM-B was initially identified as a LD-associated protein in the Chinese hamster ovary K2 cells by proteomic analysis [17]. AAM-B is classified as a putative methyltransferase. AAM-B consists of the N and C-terminal domains, juxtamembrane region, and a putative central catalytic domain for methyltransferase. It has been previously reported that N-terminal hydrophobic 28 amino acid sequence of the AAM-B is necessary to be inserted in the ER membrane and migrates from the inserted site to LDs [18]. AAM-B recruits other cellular proteins to the LDs for the formation of functional organelles [19].

Previous studies indicate that AAM-B is an LD-associated protein [17]. LD-associated proteins are important for HCV replication and production of HCV particles. We here showed that inhibition of AAM-B gene expression impaired HCV propagation. However, knockdown of AAM-B had no effect on lipid droplet formation. AAM-B interacts and colocalizes with NS4B protein. However, NS4B did not colocalize with LDs. Instead, AAM-B is predominantly localized in LD and may be involved in recruitment of NS4B protein in the proximity of LD. These data provide insights into how HCV usurps AAM-B to facilitate viral propagation.

## Materials and Methods

### Plasmid construction

Total RNAs were isolated from Huh7.5 cells by using RiboEx (GeneAll). cDNAs were synthesized by using a cDNA synthesis kit (Toyobo) according to the manufacturer’s instructions. Full-length AAM-B was amplified by a primer set: (sense, 5'-ATGAATTCTATGGAGCTTACCATCTTT-3'; antisense, 5'-TCTCTAGACTTTTCACAGCATATCCATAG-3'). The amplified products were ligated into the *Eco*RI and *Xba*I sites of the pEF6/V5-HisB (Invitrogen) plasmid. Myc-tagged NS4B plasmid has been described previously [20]. NS4B deletion mutants were constructed using following primer sets: (sense, 5'-ATGGATCCACCATGGCCTCACAACTTCCTTACATC-3'; antisense, 5'-ATGAATTCCACAGTATTGCTGCGCACACGACC-3' for M1 deletion mutant of NS4B; (sense, 5'-ATGGATCCACCATGCCCGCGATAGCATCATTGATG-3'; antisense, 5'-ATGAATTCCAGCATGGCGTAGAGCAGTCCTC-3' for M2 deletion mutant of NS4B. The PCR-amplified products were ligated into the *Bam*HI and *Eco*RI sites of the pEF6/Myc-His B plasmid. Myc-tagged core, NS3, NS5A, and NS5B plasmids were described previously [21]. siRNA-resistant mutant AAM-B was constructed by using site-directed mutagenesis kit (Stratagene) according to the manufacturer’s instruction with following primer sets: (sense, 5'-TCTTGGTGAGGTTCACTG TGATATACAATGAGCAAATGGCAAGCAAGAAG-3'; antisense, 5'-CTTCTTGCTTGCCAT TTGCTCATTGTATATCACAGTGAACCTCACCAAGA-3'.

### Cell Culture

All cell lines were grown as we previously reported [20,21]. Huh7.5 cells were grown in DMEM supplemented with 10% FBS, 1% non-essential amino acid, and 1% penicillin-streptomycin. HCV subgenomic replicon cells were grown in DMEM supplemented with 10% FBS, 1% penicillin-streptomycin, and 500 μg/ml G418.

### Antibodies

Anti-c-Myc and anti-β actin antibodies were purchased from Santa Cruz. Mouse anti-V5 antibodies were obtained from Invitrogen. Mouse anti-NS4B antibodies were purchased from Virostat. Both anti-HCV NS3 and NS5A antibodies were described previously [22].

### Generation of AAM-B stable cells

Huh7 cells stably expressing V5-AAM-B were generated by transfection of the pEF6-V5-AAM-B expression plasmid for 3 weeks in the presence of 5 μg/ml blasticidine (Invitrogen). Single positive colonies were selected by immunoblot analysis using anti-V5 monoclonal antibody. Huh7 cells transfected with an empty vector (pEF6B-V5 only) were selected as described above and were used as a control.

### Generation of HCVcc

Cell culture-derived HCV (HCVcc) was generated as describe previously [23].

### MTT assay

Huh7.5 cells were transfected with siRNAs and were incubated with 3-(4,5-dimethylthiazol-2-yl)-2,5-diphenyltetrazolium bromide (MTT) reagent (Sigma) for 2 h at 37°C. MTT assay was performed as we reported previously [22].

### RNA interference

siRNAs targeting AAM-B (AAM-B #1: 5ʹ-GGU UAA GAG CAG AGG UUU A-3ʹ; AAM-B #2: 5ʹ-AGU GUG AGC UGG CAG UUA A-3ʹ; AAM-B #3: 5ʹ-UGA UAU ACA ACG AAC AGA U-3ʹ) were purchased from Bioneer (Korea). The negative control siRNA was also obtained from Bioneer. siRNA transfection was performed by using a Lipofectamine RNAiMax reagent (Invitrogen, Carlsbad, CA) according to the manufacturer’s protocol.

### Immunoblot analysis

Cells were washed twice with phosphate-buffered saline (PBS) and were lysed in RIPA buffer (50 mM Tris-HCl [pH 7.5], 1% NP-40, 150 mM NaCl, 1 mM EDTA, 1 mM NaF, 1 mM Na_3_VO_4_, and 1 mM PMSF) for 15 min on ice, and centrifuged at 12,000 rpm for 5 min at 4°C. Equal amounts of protein collected from the supernatant were resolved by SDS-PAGE and revealed by using an ECL kit (Abfrontier).

### Quantitative real-time PCR analysis

Total RNAs were isolated from cells using RiboEX reagent (GeneAll) according to the manufacturer’s instructions. cDNAs were synthesized by using a cDNA synthesis kit (Toyobo) according to the manufacturer’ instructions. Quantitative real-time PCR (qPCR) was performed using following primers: (sense) 5'-TTA GTA TGA GAG TCG TAC AGC CTC CAG-3' and (antisense) 5'-GGC ATA GAG TGG GTT TAT CCA AGA AAG G-3' for HCV; (sense) 5'-TGA CAG CAG TCG GTT GGA GCG-3' and (antisense) 5'-GAC TTC CTG TAA CAA CGC ATC TCA TA-3' for β-actin; (sense) 5'-CAC TTA AAT CAC TCC AAA GAA GAC- 3' and (antisense) 5'-AAA TCT GAG CTG TTT AGA CTC A-3' for AAM-B. qPCR experiments were performed by using an CFX Connect real-time system (Bio-Rad Laboratories, Hercules, CA) under the following condition: 15 min at 95°C followed by 40 cycles of 95°C for 20 s, 55°C for 20 s, and 73°C for 20 s. Seventy one cycles of 10 s, with 0.5°C temperature increments from 60°C to 95°C, were used for the melting curves.

### Immunofluorescence assay

Huh7.5 cells were seeded on glass slide. At the indicated time points, cells were fixed with 4% paraformaldehyde for 30 min at room temperature. Cells were permeabilized with 0.1% triton X-100 in PBS for 10 min and blocked in 0.5% BSA for 1 h. The cells were then incubated overnight with primary antibody at 4°C. After three washes with PBS, cells were further incubated with either tetramethylrhodamine isothiocyanate (TRITC)-conjugated donkey anti-rabbit IgG or fluorescein isothiocyanate (FITC)-conjugated goat anti-mouse IgG and 1 μM of BODIPY (Invitrogen) for 1 h at room temperature. Cells were counterstained with 4′,6-diamidino-2-phenylindole (DAPI) to label nuclei. After two washes with PBS, images were processed using the Zeiss LSM 700 laser confocal microscopy system (Carl Zeiss, Inc., Thornwood, NY).

### Immunoprecipitation assay

HEK293T cells were cotransfected with V5-tagged AAM-B and Myc-tagged core, NS3, NS4B, NS5A, and NS5B, respectively. Equal amounts of DNA were transfected by adjusting with an empty vector. Total cell lysates harvested at 48 h after transfection were immunoprecipitated as we described previously [22].

### Statistical analysis

Data are presented as means ± standard deviations (SD) for at least two independent experiments. Student’s *t* test was used for statistical analysis. The asterisks in the figures indicate significant differences (*, *P*<0.05; **, *P*<0.01).

## Results

### AAM-B is required for HCV propagation

**Fig 1:**
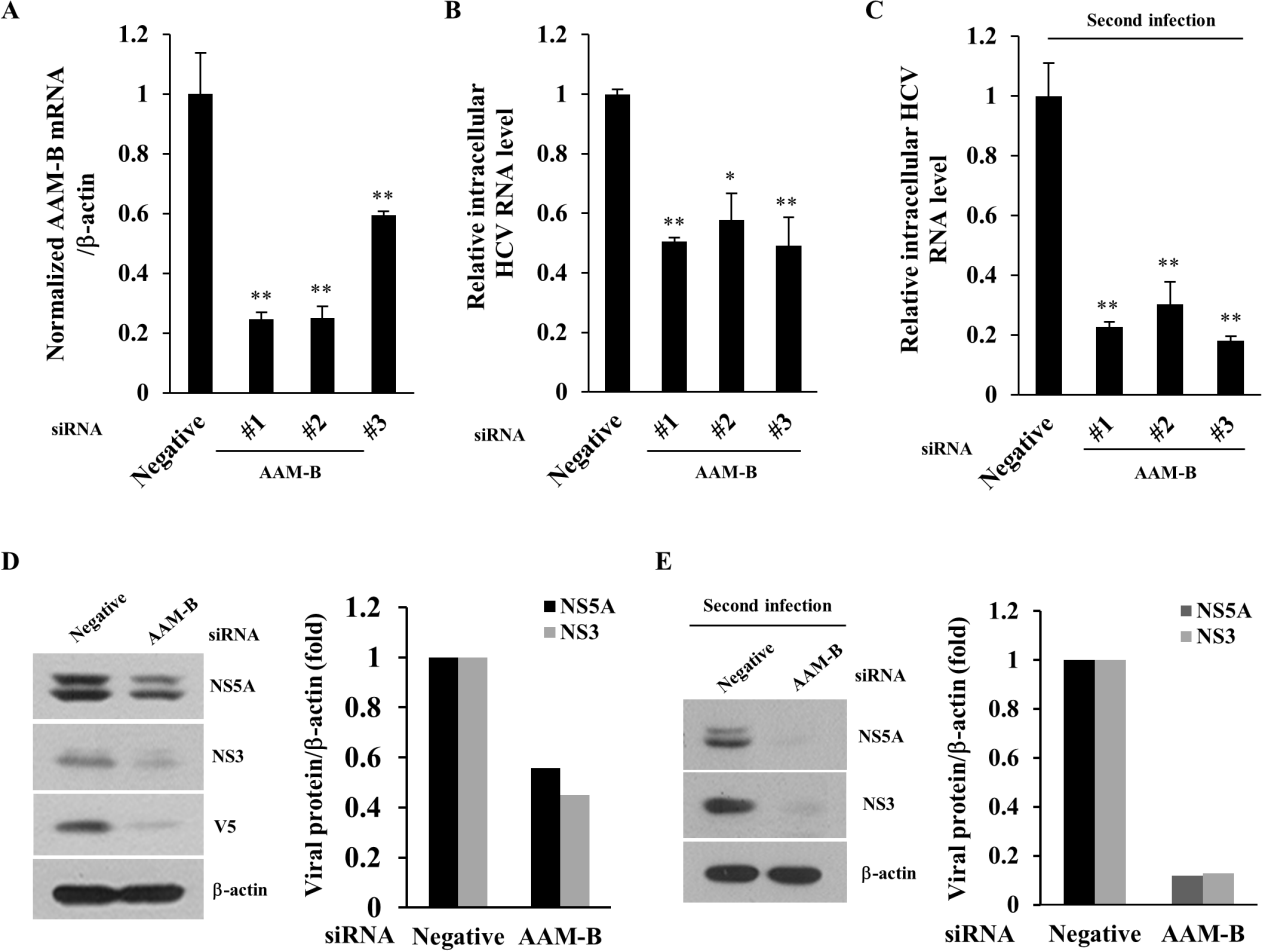
AAM-B is required for HCV propagation. (A) Huh7.5 cells were transfected with 20 nM of scrambled siRNA (Negative) or the indicated AAM-B specific siRNA constructs for 48 h and then infected with Jc1 for 4 h. At 48 h postinfection, AAM-B mRNA levels were analyzed by qPCR. (B) Using total RNAs isolated from (A), intracellular HCV RNA levels were quantified by qPCR. (C) Naïve Huh7.5 cells were infected with Jc1 harvested from the culture supernatants of (A). At 48 h postinfection, intracellular HCV RNA levels were quantified by qPCR. (D) (Left) AAM-B stable cells were transfected with the indicated siRNAs for 48 h and then infected with Jc1 for 4 h. At 48 h postinfection, total cell lysates were immunoblotted with the indicated antibodies. (Right) The band intensities of viral proteins were quantified using ImageJ software and were expressed as relative fold from the negative control. (E) (Left) Naïve Huh7.5 cells were infected with Jc1 harvested from culture supernatants of (D). At 48 h postinfection, total cell lysates were immunoblotted with the indicated antibodies. (Right) The band intensities of viral proteins were determined by using ImageJ software and were shown as relative fold from the negative control.

Host cellular lipid metabolism is essential for the replication of HCV. To identify the novel genes involved in lipid metabolism and lipid droplet formation that may play important roles in HCV propagation, we have recently performed siRNA library screening in HCVcc-infected cells [24]. siRNA that altered the HCV particle release by an average twofold compared to negative control were selected as hits. From the 114 siRNA pools targeting lipid metabolism, we identified 10 candidate genes including HMGCS1, XBP1, ARFRP1, SGPL1, ACSL1, METTL7A, FDFT1, PPAP2B, METTL7B, and SCD1. Among candidate host genes, we selected AAM-B (also known as METTL7A) and investigated its functional involvement in HCV propagation. We first verified the efficiency of each siRNA-mediated knockdown of AAM-B. Fig 1A showed that AAM-B mRNA levels were significantly decreased by each of AAM-B specific siRNA. Silencing of AAM-B expression led to significant reduction of intracellular HCV RNA (Fig 1B). Intracellular HCV RNA level was decreased by ~50% in cells transfected with #1 siRNA, ~40% with #2 siRNA, and ~45% with #3 siRNA, respectively. Knockdown of AAM-B had no effect on cell viability (data not shown), indicating that impairment of HCV replication by siRNA knockdown was not caused by cellular cytotoxicity. To examine the effect of AAM-B knockdown on HCV infectivity, naïve Huh7.5 cells were infected with Jc1 harvested from culture supernatant of primary HCV infected cells. As shown in Fig 1C, intracellular HCV RNA levels were significantly decreased by ~70 to 80%, indicating that HCV infectivity was also potently impaired by knockdown of AAM-B. Although AAM-B mRNA levels were quantified by qPCR, we were unable to detect endogenous AAM-B protein by using several sources of commercially available anti-AAM-B antibodies. To further clarify the role of AAM-B in HCV propagation, we therefore established AAM-B stable cells and then examined the effect of AAM-B knockdown on HCV protein level. Huh7 cells stably expressing AAM-B were transfected with the indicated siRNAs and then infected with Jc1. As shown in Fig 1D, HCV protein expressions were also impaired by knockdown of AAM-B. When naïve Huh 7.5 cells were infected with Jc1 harvested from culture supernatants of Fig 1D, HCV protein expressions were dramatically decreased, further confirming that HCV infectivity was impaired by knockdown of AAM-B (Fig 1E). These results indicate that AAM-B may be an important cellular factor required for HCV propagation.

### AAM-B is not required for the replication step of the HCV life cycle

**Fig 2:**
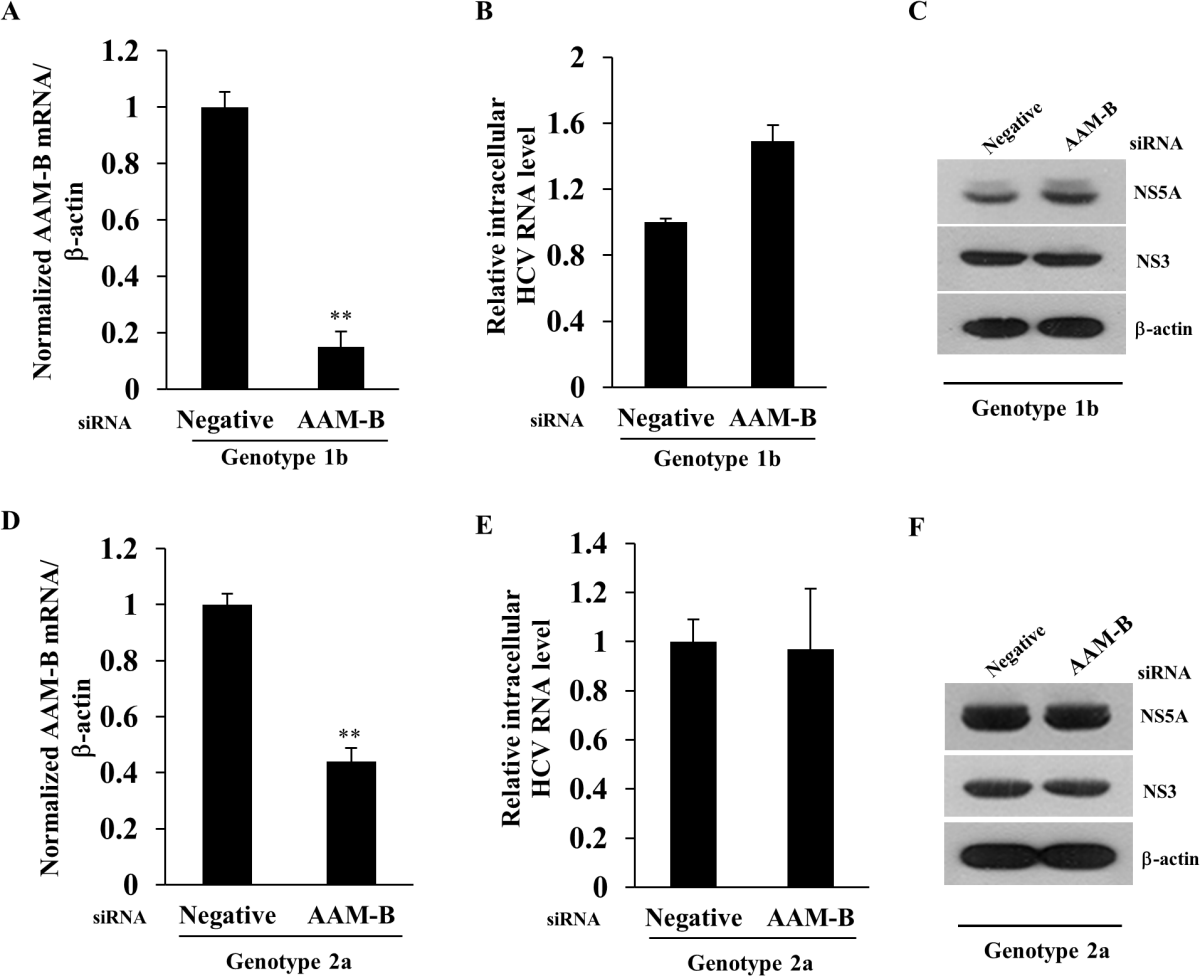
AAM-B is not required in the replication step of the HCV life cycle. Huh7 cells harboring HCV subgenomic replicon derived from genotype 1b were transfected with the indicated siRNAs for 72 h. Both AAM-B mRNA level (A) and intracellular HCV RNA level (B) were quantified by qPCR. (C) Protein expression levels were analyzed by immunoblot analysis using the indicated antibodies. (D) Huh6 cells harboring HCV subgenomic replicon derived from genotype 2a were transfected with the indicated siRNAs for 72 h. Both AAM-B mRNA level (D) and intracellular HCV RNA level (E) were quantified by qPCR. The asterisks indicate significant difference (**, *p*<0.01) from the value for the negative control. (F) Protein expression levels in HCV replicon cells of genotype 2a were analyzed by immunoblotting with the indicated antibodies.

To examine which step of the HCV life cycle required for AAM-B, we first explored the possible involvement of AAM-B in HCV replication step using replicon system. When AAM-B was sufficiently knockdown (Fig 2A), neither intracellular HCV RNA level (Fig 2B) nor HCV protein expression level (Fig 2C) was altered in subgenomic replicon cells derived from genotype 1b. We further verified that knockdown of AAM-B (Fig 2D) exerted no effect on HCV RNA (Fig 2E) and HCV protein expression (Fig 2F) levels in subgenomic replicon cells harboring genotype 2a. These data indicate that AAM-B is not involved in the replication step of the HCV life cycle.

### AAM-B is involved in the assembly and release stages of HCV life cycle

**Fig 3:**
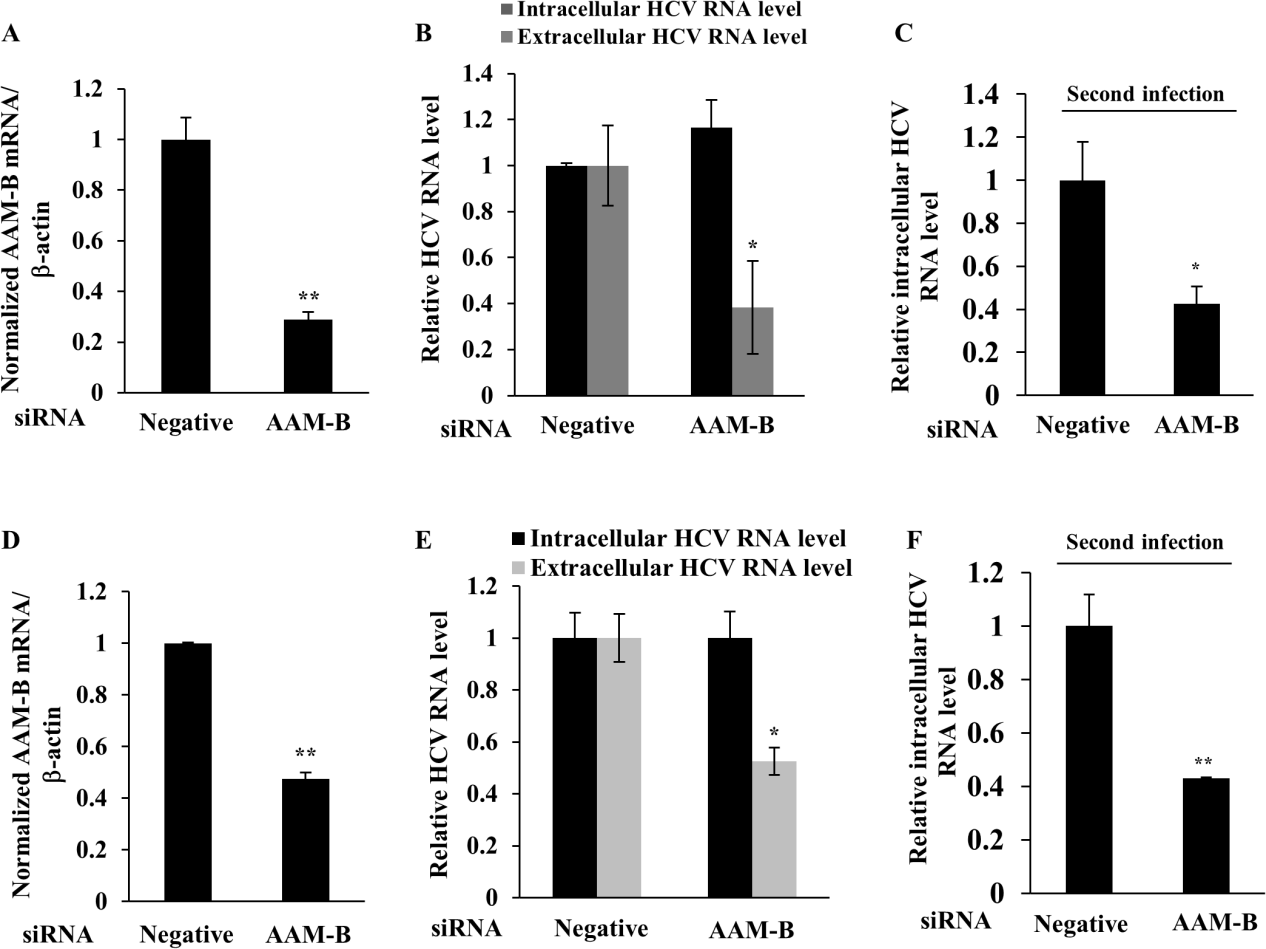
AAM-B is involved in the virion assembly and release stage of the HCV life cycle. (A, B) Huh7.5 cells were electroporated with in vitro transcribed Jc1 RNA for 24 h and then transfected with the indicated siRNAs. At 48 h after transfection, both AAM-B mRNA level (A) and HCV RNA levels (B) were quantified by qPCR. (C) Naïve Huh7.5 cells were infected with culture supernatant harvested from (A). At 48 h postinfection, intracellular HCV RNA level was quantified by qPCR. (D) AAM-B stable cells were infected with Jc1 for 4 h. At 48 h postinfection, the cells were transfected with the indicated siRNAs. At 48 h after siRNA transfection, both AAM-B mRNA level (D) and HCV RNA levels (E) were quantified by qPCR. (F) Naïve Huh7 cells were infected with culture supernatant harvested from (D). At 48 h postinfection, intracellular HCV RNA level was quantified by qPCR. The asterisks indicate significant differences (*, *p*<0.05;**, *p*<0.01) from the value for the negative control.

To further investigate the role of AAM-B in HCV propagation, Huh7.5 cells were electroporated with in vitro transcribed Jc1 RNA. At 24 h after RNA electroporation, cells were further transfected with either negative or AAM-B-specific siRNAs. Although AAM-B mRNA level was significantly decreased by knockdown by siRNA treatment (Fig 3A), intracellular HCV RNA level was unaffected by knockdown of AAM-B (Fig 3B). However, extracellular HCV RNA level was decreased by 60% in AAM-B knockdown cells. To confirm this result, naïve Huh7.5 cells were infected with Jc1 harvested from culture supernatant of Fig 3A. As shown in Fig 3C, intracellular HCV RNA level was decreased by ~60% in AAM-B knockdown cells, indicating that virion release was impaired in AAM-B knockdown cells. To further verify these results, we investigated the effect of AAM-B knockdown on HCV propagation using AAM-B stable cells. AAM-B stable cells were infected with Jc1 and then transfected with either negative or AAM-B-specific siRNAs. Likewise, intracellular HCV RNA level was unaltered, whereas both extracellular HCV RNA level and HCV infectivity were significantly decreased in AAM-B knockdown cells (Fig 3D–3F). These data suggest that AAM-B may be involved in the assembly and release stages of HCV life cycle and thus play a crucial role in HCV propagation.

### AAM-B is required for the HCV particle production without affecting lipid droplet formation

**Fig 4:**
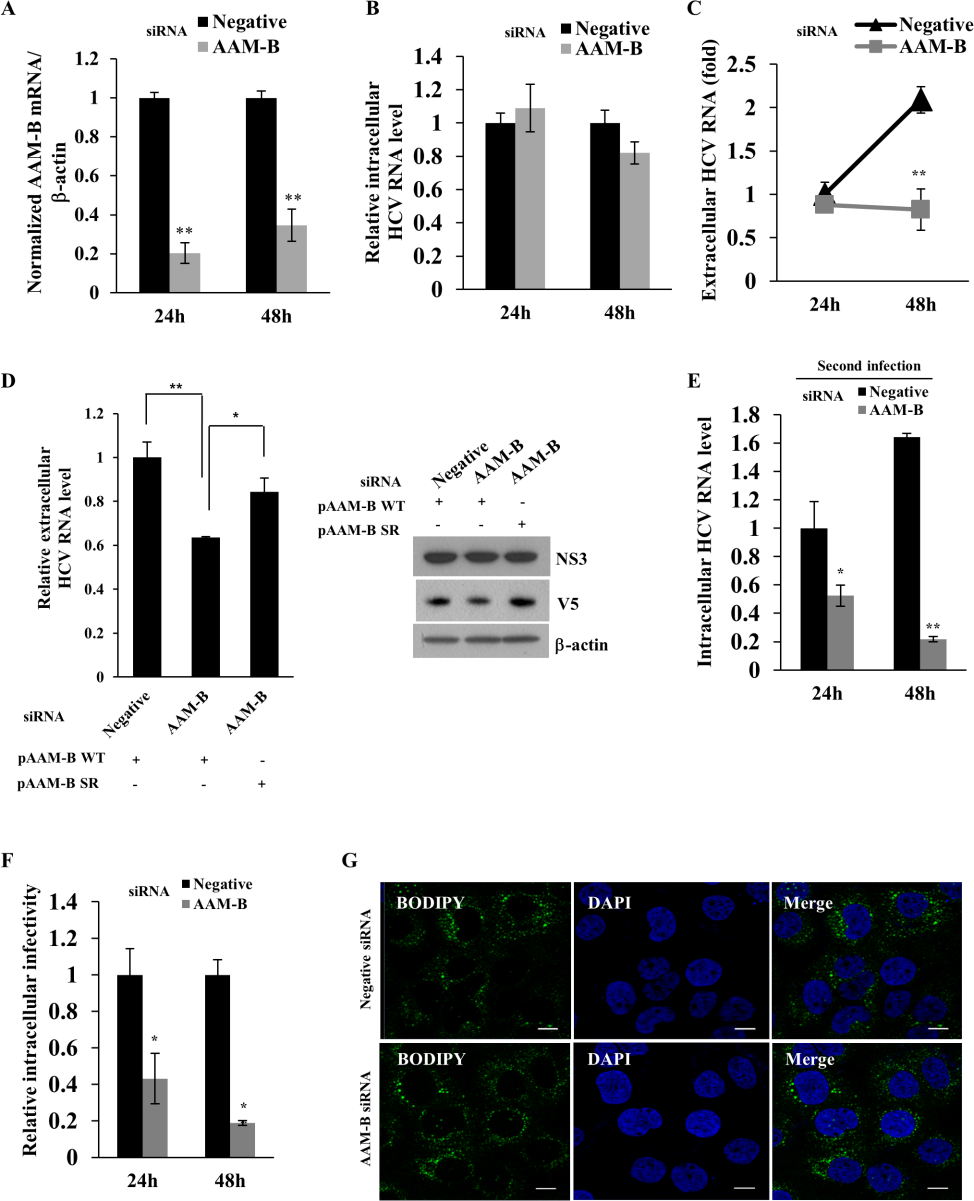
AAM-B is required for the virion production without affecting lipid droplet formation. (A) Huh7 cells were infected with Jc1 for 4 h. At 48 h postinfection, cells were further transfected with the indicated siRNAs. At 24 h after siRNA transfection, the culture medium was replaced with fresh medium and cells were further grown for the indicated time points. AAM-B mRNA levels (A), intracellular HCV RNA levels (B), and extracellular HCV RNA levels (C) were quantified by qPCR. (D) (Left) Huh7 cells were infected with Jc1 for 4 h. At 48 h postinfection, cells were further transfected with the indicated siRNAs. At 24 h after siRNA transfection, the cells were transfected with plasmid expressing either wild-type AAM-B or siRNA resistant mutant AAM-B. At 48 h after plasmid transfection, extracellular HCV RNAs were quantified by qPCR. pAAM-B WT, V5-tagged wild-type AAM-B; pAAM-B SR, V5-tagged siRNA resistant mutant AAM-B. (Right) Huh7 cells were treated as described in the left panel and protein expressions were analyzed by immunoblot analysis using the indicated antibodies. (E) Naïve Huh7 cells were infected with culture supernatant harvested from (A) for 4 h. At 48 h postinfection, intracellular HCV RNA levels were analyzed by qPCR. (F) Huh7 cells were infected with Jc1 for 4 h. At 48 h postinfection, the cells were transfected with either negative or AAM-B specific siRNAs. At the indicated time points, the cells were harvested and washed twice with PBS. The cells were subjected to three consecutive freeze-thaw cycles and then centrifuged for 30 min in a microfuge. Supernatant was used as intracellular Jc1. Naïve Huh7 cells were infected with supernatant for 4 h. At 48 h postinfection, relative intracellular HCV infectivity was determined by qPCR. (G) Huh7 cells were infected with Jc1 for 4 h. At 48 h postinfection, cells were further transfected with the indicated siRNAs. The cells were fixed and incubated with BODIPY for 1 h at 37°C. Cells were counterstained with 4',6-diamidino-2-phenylindole(DAPI) to label nuclei. Immunofluorescence images were processed using the Zeiss LSM 700 laser confocal microscopy system. Bars, 10μm.

To further clarify whether AAM-B was involved in the production of HCV particles, Huh7 cells were infected with Jc1 and then transfected with either negative or AAM-B-specific siRNAs. After 24 h after transfection, culture medium was replaced with fresh medium and then both cells and culture supernatants were harvested at the indicated time points. Fig 4A showed that silencing of AAM-B sufficiently suppressed mRNA level of AAM-B. Nevertheless, intracellular HCV RNA level was unaffected in AAM-B knockdown cells (Fig 4B). Meanwhile, extracellular HCV RNA level was significantly decreased at 48 h after knockdown of AAM-B (Fig 4C). To rule out the off-target effect of AAM-B siRNA, we generated a siRNA-resistant AAM-B mutant. As shown in Fig 4D, exogenous expression of a siRNA-resistant AAM-B mutant, but not of wild-type AAM-B, rescued the silencing effect of AAM-B on extracellular HCV RNA level without affecting HCV protein expression. To investigate whether HCV infectivity was affected by knockdown of AAM-B, naïve Huh7 cells were infected with HCV harvested from culture supernatants of Fig 4A and then intracellular HCV RNA level was determined by qPCR. As shown in Fig 4E, infectivity of released HCV particles was significantly decreased at the indicated time points in AAM-B knockdown cells. To further investigate the intracellular infectivity of HCV, cells inoculated with released HCV particles were subjected to repetitive freeze-thaw cycles, and then supernatants were used to infect naïve Huh7 cells. As shown in Fig 4F, intracellular HCV infectivity was significantly decreased in AAM-B knockdown cells. These data indicate that AAM-B is required for the production of infectious HCV particles. Since LD is an important organelle for hepatitis C virus production [11], we explored the possible involvement of AAM-B in LD formation. As shown in Fig 4G, knockdown of AAM-B did not alter the formation of LD, indicating that AAM-B might regulate HCV propagation without affecting LD formation.

### AAM-B interacts with HCV NS4B protein

**Fig 5:**
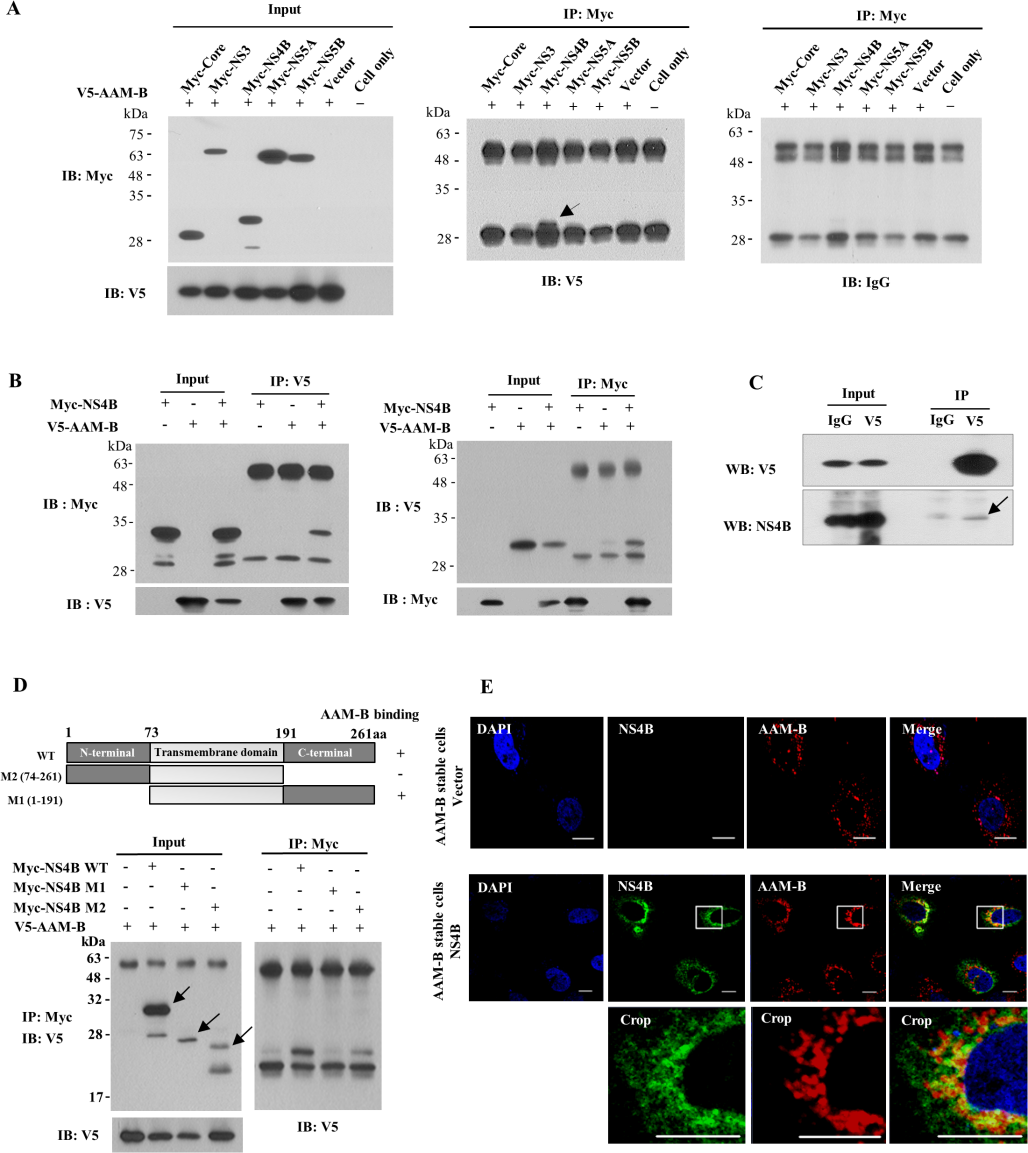
AAM-B interacts with HCV NS4B protein. (A) HEK293T cells were cotransfected with V5-tagged AAM-B plasmid and Myc-tagged HCV protein as indicated. Protein expressions of input plasmids were confirmed by immunoblot analysis using either anti-Myc antibody (Left, upper) or anti-V5 antibody (Left, lower). (Right) Total cell lysates were immunoprecipitated with anti-Myc antibody and then bound proteins were immunoblotted with anti-V5 antibody. The arrow indicates the interacting band. (B) (Left) HEK293T cells were cotransfected with V5-tagged AAM-B plasmid and Myc-tagged NS4B (genotype 1b) expressing plasmid. At 48 h after transfection, total cell lysates were immunoprecipitated with anti-V5 antibody and then bound proteins were immunoblotted with anti-Myc antibody. (Right) The same cell lysates were immunoprecipitated with anti-Myc antibody and bound proteins were immunoblotted with anti-V5 antibody. (C) Huh7 cells harboring subgenomic replicon derived from genotype 1b were transfected with V5-tagged AAM-B expression plasmid. Total cell lysates were immunoprecipitated with rabbit anti-V5 antibody and then bound proteins were immunoblotted with either mouse anti-V5 antibody (left) or mouse anti-NS4B (right). The arrow indicates the interacting band. (D) (Upper) Schematic representation of both wild-type and mutant constructs of NS4B protein. (Lower) HEK293T cells were cotransfected with V5-tagged AAM-B and Myc-tagged mutant constructs of NS4B. Total cell lysates harvested at 48 h after transfection were immunoprecipitated with anti-Myc antibody and bound proteins were immunoblotted with anti-V5 antibody. Both wild-type and mutant protein expressions of NS4B were verified by immunoprecipitation and immunoblot analysis using anti-Myc antibody (arrows). (E) AAM-B stable cells were transiently transfected with either vector or NS4B expression plasmid. At 48 h after transfection, cells were fixed in 4% paraformaldehyde. Cells were washed twice with PBS and then incubated with either anti-NS4B or anti-AAM-B antibodies for 1 h in room temperature. Immunofluorescence staining was performed by using either FITC-conjugated or TRICT-conjugated secondary antibodies. The inset in each panel shows enlarged image of the area marked in a white square. Bars, 10 μm.

To investigate whether functional involvement of AAM-B in HCV propagation was mediated through virus-host protein interaction, we cotransfected HEK293T cells with V5-tagged AAM-B plasmid and various Myc-tagged HCV protein expression plasmid. At 48 h after transfection, total cellular proteins were immunoprecipitated with an anti-Myc antibody and then bound proteins were immunoblotted with an anti-V5 antibody. As shown in Fig 5A, AAM-B interacted with HCV NS4B protein but not with other viral proteins. To further confirm this result, HEK293T cells cotransfected with Myc-tagged NS4B and V5-tagged AAM-B plasmid were immunoprecipitated with an anti-V5 antibody and bound proteins were immunoblotted with an anti-Myc antibody. Fig 5B showed that AAM-B specifically interacted with NS4B (left panel). Reciprocal experiment further verified protein interplay between AAM-B and NS4B (Fig 5B, right panel). To further verify this interaction in the context of HCV replication, Huh7 cells harboring HCV subgenomic replicon derived from genotype 1b were transfected with V5-tagged AAM-B expression plasmid. As demonstrated in Fig 5C, AAM-B specifically interacted with NS4B protein in HCV RNA replicating cells. To delineate the binding region of AAM-B in NS4B, we constructed NS4B deletion mutants (Fig 5D, upper panel). HEK293T cells were cotransfected with AAM-B and various NS4B deletion constructs, and then binding domain was determined by immunoprecipitation assay. As shown in Fig 5D (lower panel), AAM-B interacted with C-terminal region of NS4B protein. These data suggested that AAM-B might colocalize with NS4B. To investigate this possibility, AAM-B stable cells were transiently transfected with either vector or NS4B expression plasmid and examined for the subcellular localization by confocal microscopy. As shown in Fig 5E, both NS4B and AAM-B proteins localized in the cytoplasm, and dual staining showed colocalization as yellow fluorescence. Manders’ overlap coefficient was 0.834 ± 0.113, verifying the colocalization of NS4B and AAM-B proteins. Since NS4B-induced membrane structures may not be equivalent to those observed when other HCV nonstructural proteins are co-expressed, we performed immunofluorescence assay using Huh7 cells harboring HCV subgenomic replicon. However, staining intensity of NS4B particularly in immunofluorescence assay was so weak that colocalization of AAM-B and NS4B was hardly detectable in the context of HCV replication.

### AAM-B is predominantly localized in LD and may be involved in recruitment of NS4B protein in the proximity of LD

**Fig 6:**
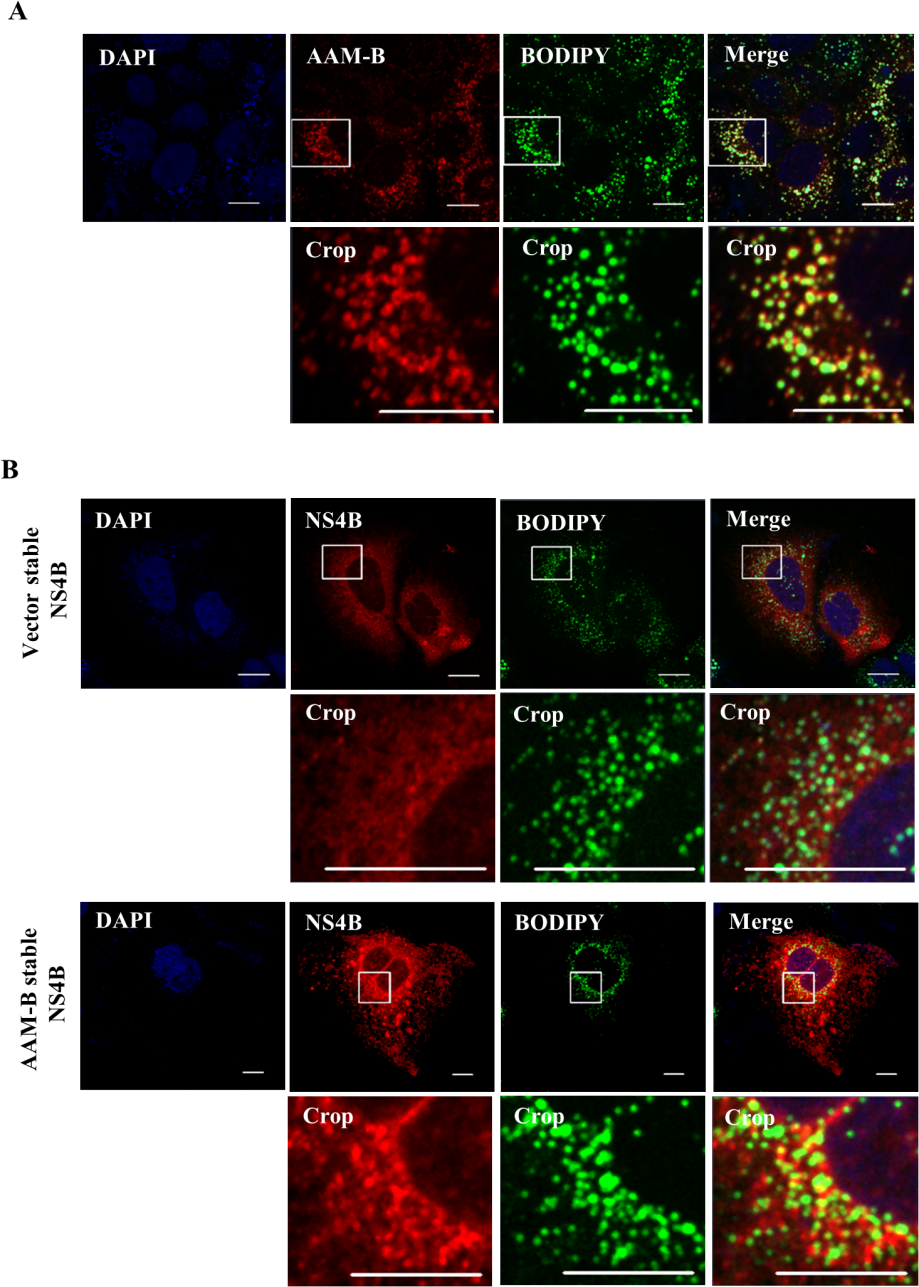
AAM-B colocalizes with NS4B in the proximity to the lipid droplets. (A) AAM-B stable cells fixed with paraformaldehyde were incubated with anti-V5 antibody to detect AAM-B and BODIPY. AAM-B colocalized with LD in the cytoplasm. Bars, 10 μm. (B) Either vector stable or AAM-B stable cells were transiently transfected with NS4B plasmid. At 48 h after transfection, cells were fixed in 4% paraformaldehyde and incubated with anti-NS4B antibody and BODIPY for 1 h at 37°C. Samples were analyzed for immunofluorescence staining using the LSM 700 laser confocal microscopy system. Cells were counterstained with DAPI to label nuclei. The insets in the panels show enlarged views of the areas marked in white squares. Bars, 10 μm.

LD is an essential organelle for the production of HCV particles [10]. It has been reported that coat protein localized on the surface of the LD is involved in HCV particle assembly [13–16]. By proteomic analysis, AAM-B was identified as LD-associated protein in the Chinese hamster ovary K2 cells [17]. It has been shown that AAM-B is inserted in the ER membrane and migrates from the inserted site to LD [18]. Moreover, AAM-B has ability to drag and recruit other proteins to the LD [19]. For this reason, we examined the colocalization of AAM-B and LD. Indeed, AAM-B colocalized with LD in the cytoplasm (Fig 6A). It was noteworthy that LD appeared to be encircled by AAM-B. Since AAM-B interacts with NS4B, we explored whether NS4B could colocalize with LD. For this purpose, either vector stable or AAM-B stable cells were transiently transfected with NS4B expression plasmid and examined colocalization by immunofluorescence assay. As shown in Fig 6B, NS4B localized in the vicinity of LDs in AAM-B stable cells but not in vector stable cells. This indicates that AAM-B might be involved in recruitment of NS4B to sites in close proximity to LDs to facilitate HCV propagation.

## Discussion

HCV exploits lipid metabolism for its own propagation [9]. In the present study, we identified AAM-B as a host gene that was required for the HCV propagation. AAM-B is a putative methyltransferase which is anchored in ER membrane. AAM-B is a LD-associated protein [19]. N-terminal hydrophobic 28 aa sequence of the AAM-B is required to be inserted in the ER membrane and migrates from the inserted site to LDs [18]. AAM-B recruits other proteins to the LDs to make functional organelles [19]. LD is an essential organelle for the production of HCV particles [10]. It has been reported that the LD-associated proteins are required for HCV replication and the production of HCV particles [13–16]. The surface of the LD is coated with a variety of proteins which play an important role in the regulation of LD maintenance and function [11–12]. It has been previously reported that Rab18, an LD-associated protein, interacts with HCV NS5A and regulates HCV replication and possibly production of infectious HCV particles by promoting the physical interaction between the HCV and LD [13]. In addition, TIP47 which is also localized in the surface of LD regulates HCV replication and particle release [14–16]. However, the detailed mechanism of LD involvement in HCV particle production has not been fully understood.

We have shown that siRNA-mediated knockdown of AAM-B impaired HCV propagation. Silencing of AAM-B resulted in significant decrease of HCV particles assembly and release without affecting intracellular HCV RNA and protein expression levels. We confirmed that HCV replication levels in subgenomic replicon cells derived from genotype 1b and 2a were unaltered in AAM-B knockdown cells. We further verified that knockdown of AAM-B dramatically decreased both extracellular HCV RNA and infectivity levels, but not intracellular HCV RNA, in Jc1 RNA electroporated Huh7.5 cells and in Jc1 infected AAM-B stable cells. To further clarify which step of HCV life cycle required for AAM-B, Huh7 cells were infected with Jc1 and then transfected with AAM-B siRNA. Likewise, intracellular HCV RNA level was not changed in AAM-B knockdown cells. We further confirmed that knockdown of AAM-B displayed no effect on HCV entry (data not shown). However, both extracellular HCV RNA and infectivity levels in released HCV particles were significantly decreased in AAM-B knockdown cells. These results suggest that AAM-B is involved in the assembly and release stages of HCV life cycle. Indeed, the infectivity of intracellular HCV particles was significantly decreased in AAM-B knockdown cells.

We next investigated whether knockdown of AAM-B could alter the LD formation. Immunofluorescence data indicated that AAM-B modulated HCV production without affecting lipid droplet formation. We therefore explored the possible involvement of viral protein in AAM-B-mediated HCV propagation. We demonstrated that AAM-B specifically interacted with HCV NS4B protein. Moreover, AAM-B colocalized with NS4B protein. We showed that NS4B and LD were not colocalized in the cytoplasm. Rather, NS4B localized in the vicinity of LDs. Since AAM-B is a LD-associated protein, this suggests that AAM-B may recruit NS4B to sites in close proximity to LDs to make functional organelles for the efficient production of particles. Growing evidences suggest that NS4B plays an important role in the production of HCV particles as well as formation of membranous web [25–27]. Amino acid substitution at N216A in C-terminal region of NS4B increased production of JHF1 virus without affecting RNA replication [26]. Swapping the C-terminal region of NS4B in JFH1 with sequences of either Con1 or H77 decreased production of HCV with no effect on RNA replication. The C-terminal region of NS4B is crucial for genome encapsidation and thus NS4B may regulate host proteins for HCV production [27]. We also demonstrated that AAM-B interacted with the C-terminal region of NS4B and involved in production of HCV. However, the detailed molecular mechanism of how AAM-B regulates HCV particle production needs further investigation. Collectively, our current study highlights the crucial role of AAM-B in HCV propagation and thus AAM-B may be an attractive target for therapeutic intervention.
